# Improvement of respiratory symptoms and health‐related quality of life with peramivir in influenza patients with chronic respiratory disease: Additional outcomes of a randomized, open‐label study

**DOI:** 10.1111/irv.12835

**Published:** 2021-03-08

**Authors:** Motokazu Kato, Yutaka Saisho, Hiroshi Tanaka, Takuma Bando

**Affiliations:** ^1^ Chest Disease Clinical and Research Institute Kishiwada City Hospital Osaka Japan; ^2^ Shionogi & Co., Ltd. Osaka Japan; ^3^ NPO Sapporo Cough Asthma and Allergy Center Sapporo Japan; ^4^ Bando Internal Medicine Clinic Hakusan Japan; ^5^ Present address: Respiratory Institute Kamei Hospital Osaka Japan

**Keywords:** asthma, COPD assessment test, cough, influenza, peramivir

## Abstract

**Background:**

To compare peramivir 300 mg single‐dose, peramivir 600 mg repeat‐dose, and oseltamivir effects on health‐related quality of life, including respiratory symptoms and general conditions, time to symptom alleviation, time to fever resolution, incidence of exacerbations, and virus titer, in influenza patients with chronic respiratory disease.

**Methods:**

We report additional outcomes from a 2‐week, multicenter, randomized, open‐label study in Japan (UMIN000030118). Influenza patients with chronic respiratory disease received intravenous peramivir (300 mg single‐dose [n = 66], 600 mg repeat‐dose [600 mg/d of 2 consecutive days; n = 70]) or oral oseltamivir (75 mg twice daily, 5 days; n = 72). The principal endpoint of this analysis was change from baseline to Day 14 at each time point in Chronic Obstructive Pulmonary Disease Assessment Test (CAT) scores.

**Results:**

Both peramivir regimens reduced total CAT score at Day 3 more than oseltamivir (peramivir 600 mg vs oseltamivir, *P* = .0032; peramivir 300 mg vs oseltamivir, *P* = .0203). Cough/phlegm CAT scores were most improved with peramivir 600 mg. Median time to alleviation of three respiratory symptoms was longer with peramivir 600 mg (68.9 hours) than with peramivir 300 mg (50.6 hours, hazard ratio [HR] 1.57; *P* = .0191) and shorter with peramivir 300 mg than oseltamivir (78.8 hours, HR 0.62; *P* = .0141). Alleviation of seven influenza symptoms and fever resolution was shortest with peramivir 300 mg.

**Conclusions:**

Rapid improvement in CAT score, including cough, and shorter time to alleviation of respiratory symptoms associated with peramivir is of potential benefit to patients with chronic respiratory disease.

## 1 INTRODUCTION

Influenza virus infection is associated with increased morbidity^1^ and mortality^2^, ^3^ and can also trigger exacerbations of respiratory diseases such as asthma and/or chronic obstructive pulmonary disease (COPD).^4^, ^5^ Influenza patients with existing chronic respiratory disease often experience triggered asthma attacks^6^ and deterioration of lung function and need longer to recover.^4^ Influenza infection induces various cytokines and an imbalance of immune homeostasis in the respiratory system^6^ and may damage bronchial epithelial cells.^4^, ^7^

The alleviation of cough is particularly important for improving health‐related quality of life (HRQoL) in influenza patients with chronic respiratory disease. Cough is a common influenza symptom associated with respiratory tract infections and is stimulated by viral or bacterial components.^7^ Generally, patients with influenza have cough symptoms that resolve within a week, but occasionally cough persists for more than 3 weeks or can become chronic (>8 weeks).^7^, ^8^ Patients with chronic respiratory disease who have influenza may develop persistent or chronic cough.^9^, ^10^ Preventing the prolongation of cough by administration of effective treatment is required for the improvement of HRQoL.

The clinical effectiveness of peramivir, especially rapid alleviation of symptoms, has been demonstrated.^11^ Peramivir is a neuraminidase inhibitor (NAI) that reduces virus titer more rapidly than oseltamivir and elicits more rapid alleviation of fever and influenza‐related symptoms compared with other NAIs^12^, ^13^, ^14^ or placebo.^15^, ^16^ Most studies have assessed the duration of fever, alleviation of influenza symptoms, or reduction of virus titer as measures of efficacy.^11^, ^12^, ^13^, ^14^, ^17^, ^18^ However, no studies have assessed cough as a separate symptom or used the COPD Assessment Test (CAT) as an evaluation of HRQoL in influenza patients with chronic respiratory disease. The CAT is a validated, short (eight‐item), patient self‐evaluated questionnaire that can be used in routine clinical practice^19^ to measure the health status of patients with COPD or other respiratory disease (eg, bronchiectasis, asthma).^20^, ^21^

We recently reported the results of a multicenter, randomized, open‐label, controlled study in Japan in which a single 300‐mg dose of intravenous peramivir was more effective and faster to alleviate respiratory symptoms than oral oseltamivir in patients with chronic respiratory disease (primarily asthma) who had influenza.^22^ Peramivir 300 mg single‐dose, as well as 600 mg repeat‐dose, was well tolerated in these patients. The main objective of the additional analyses reported here was to compare the effect of peramivir 300 mg single‐dose, peramivir 600 mg repeat‐dose, and oseltamivir on the mean change from baseline to Day 3 in CAT score (as an assessment of HRQoL), including respiratory symptoms and general conditions. Other outcomes included the severity score and time to alleviation of influenza symptoms, time to resolution of fever, incidence rate of exacerbation events, and virus titer by subgroup in influenza patients with chronic respiratory disease.

## 2 METHODS

### 2.1 Study design

Here, we report additional outcomes from a 2‐week, multicenter, randomized, open‐label study conducted at 50 sites in Japan from October 2017 to February 2019.^22^ The study was conducted in accordance with the Declaration of Helsinki, Ethical Guidelines for Medical and Health Research Involving Human Subjects, and the revised 2017 Clinical Trials Act. The protocol was approved by local ethical review boards. All patients gave written informed consent. The study was registered at https://www.umin.ac.jp/ctr/index.htm (UMIN000030118).

### 2.2 Study population

Full study details have been previously described.^22^ Key inclusion criteria were male or female inpatients or outpatients aged 16‐79 years; diagnosis of influenza; total score of ≥ 3 for three respiratory symptoms (cough, sore throat, nasal congestion), including ≥ 1 for cough (four‐grade scoring system: 0, no symptoms; 1, mild; 2, moderate; 3, severe); ≥1 general symptom (headache, muscle or joint pain, heat or chills, and fatigue) with score ≥ 2; maximum body temperature ≥ 37.5°C for ≥ 12 hours before screening; and undergoing treatment for a chronic respiratory disease (bronchial asthma, pulmonary fibrosis, or COPD, including diseases with emphysematous changes on chest computed tomography). Patients with chronic respiratory failure who had been under ventilator management, diabetes with glycated hemoglobin A1c ≥ 10% within 4 weeks before screening, or previous treatment with an NAI, amantadine hydrochloride, or baloxavir marboxil within the last 7 days were excluded.

### 2.3 Randomization and treatment

Eligible patients were randomized (1:1:1), using the minimization method and stratified by total score of respiratory symptoms and underlying respiratory disease, to intravenous peramivir 600 mg repeat‐dose (peramivir 600 mg for 2 days), intravenous peramivir 300 mg single‐dose (peramivir 300 mg for 1 day), or oral oseltamivir (oseltamivir 75 mg twice daily for 5 days).

### 2.4 Outcome measures

The primary endpoint of the overall study was cumulative area of time vs symptoms (CATVS), assessed using an index area under the curve of the total score of three respiratory symptoms for 2 weeks.^22^ The main efficacy endpoint in this report was the mean change from baseline to Day 14, evaluated at each time point, in total score and individual item scores of the CAT. The CAT comprises eight items (cough, phlegm, chest tightness, breathlessness, activities, confidence, sleep, and energy), each scored from 0 (no symptom, best condition) to 5 (worst/severe condition), with total score from 0 to 40.

Other endpoints were also assessed. The mean change from baseline over Days 2‐14 in the total score (using the four‐grade severity system) of seven influenza symptoms, including three respiratory symptoms and four systemic symptoms, was assessed by patient diary. The time to alleviation of seven influenza symptoms and three respiratory symptoms was defined as being when scores for all symptoms were 0 (no symptoms) or 1 (mild) for ≥21.5 hours. When data were missing, symptoms were scored as 2 (moderate) or 3 (severe) and considered “not alleviated.” When cough was preexisting (ie, before influenza infection) and patients determined it was worsening at baseline (before drug administration), we defined cough as alleviated when the severity improved by ≥ 1 level (eg, change from 3 to 2, 1, or 0). When cough was preexisting and patients determined it was not worsening at baseline, we defined cough as alleviated when the severity was maintained. For time to resolution of fever, patients measured and recorded their axillary temperature four times a day (morning, noon, evening, and before sleep) from Day 1 to Day 3, and twice a day (morning and evening) from Day 4 to Day 14. The time to recover to normal axillary temperature (<37°C) from first administration of study drug was assessed. Missing values were not replaced. The incidence of exacerbation events, defined as when a respiratory‐related event (asthma attack, dyspnea and cyanosis, pneumonia, etc) needed additional treatment (additional or increase of steroid or bronchodilator, oxygen inhalation or intubation, change to hospitalization or intensive care unit, etc), occurring within 2 weeks from the first administration of study drug, was calculated. Physicians reported exacerbation events, the reasons, additional medication dosage, and respiratory function parameters. Serious events and drug‐induced pneumonia were reported as adverse events. Complications associated with influenza infection, such as otitis and sinusitis, or mild exacerbations such as increased breath, cough, or phlegm that overlapped with influenza symptoms, were not evaluated as exacerbations. The mean change from baseline at Days 2, 3, and 14 in virus titer, measured as described previously^22^ by influenza virus type and chronic respiratory disease, was calculated.

### 2.5 Statistical analysis

The planned sample size was 210 patients (70 patients in each treatment group). All randomized patients who received at least one dose of study drug were included in the analyses as the intent‐to‐treat (ITT) population. All pairwise comparisons were conducted between the three treatment groups (ie, peramivir 600 mg vs peramivir 300 mg, peramivir 600 mg vs oseltamivir, or peramivir 300 mg vs oseltamivir). Differences between groups in the total and individual item scores of CAT, or the mean changes from baseline in the total score of seven influenza symptoms, at every 24 hours were compared using a linear model with intra‐patient correlations between time points. The linear model assumed that the CAT score or the total score of seven symptoms at each time point including baseline was equal between the three treatment groups. We presumed intra‐patient correlations between time points assuming no structure, with treatment group, time point, interaction between group and time point, and chronic respiratory disease as explanatory variables. Degrees of freedom were adjusted using Kenward and Roger approximation.

For time to alleviation of seven influenza symptoms, three respiratory symptoms, and resolution of fever, hazard ratios (HRs) and their 95% confidence intervals (CIs) were calculated using a Cox proportional hazard model, with prognostic factors used for randomization (total score of three respiratory symptoms [≥5, <5]; underlying respiratory disease [bronchial asthma, COPD, or pulmonary fibrosis]) as covariates. Tied events were handled by calculating partial likelihood using the Efron method. Time to alleviation of symptoms and resolution of fever was estimated using a Kaplan‐Meier survival curve, and the median and 95% CI were calculated by the Greenwood method. Restricted mean survival time (RMST) and 95% CI from baseline to Day 14 were calculated in each treatment group, and all pairwise comparisons were conducted. The incidence of exacerbation events within 2 weeks after first study drug administration was calculated, and 95% CIs were determined using the Clopper‐Pearson method. Incidence rates were compared between the three treatment groups using a Mantel‐Haenszel test with stratified prognostic factors. Mean change from baseline at Days 2, 3, and 14 in virus titer by influenza type and chronic respiratory disease was compared between the three treatment groups using a van Elteren test with stratified prognostic factors. All analyses were conducted using SAS version 9.3 or higher (SAS Institute Japan Ltd.).

## 3 RESULTS

### 3.1 Demographic and baseline clinical characteristics

A total of 214 patients were randomized, of whom 70 patients in the peramivir 600 mg repeat‐dose group, 66 patients in the peramivir 300 mg single‐dose group, and 72 patients in the oseltamivir group were included in the ITT population (Table 1).^22^ Demographics, clinical characteristics, CAT score, and virus titer at baseline were well balanced between the treatment groups (Table 1). Approximately 90% of patients had comorbid bronchial asthma, 60% had a total score of three respiratory symptoms ≥5, and 60% were infected with influenza type A.

**TABLE 1:** Summary of baseline demographics and disease characteristics (ITT population)

Characteristic	Peramivir 600 mg N = 70	Peramivir 300 mg N = 66	Oseltamivir N = 72	*P*‐value
Age				
<65 years	51 (72.9)	53 (80.3)	56 (77.8)	.5873^a^
≥65 years	19 (27.1)	13 (19.7)	16 (22.2)	
Sex				
Male	35 (50.0)	30 (45.5)	28 (38.9)	.4287^a^
Female	35 (50.0)	36 (54.5)	44 (61.1)	
Smoking status				
Never	46 (65.7)	42 (63.6)	45 (62.5)	.9138^b^
Former smoker	15 (21.4)	13 (19.7)	23 (31.9)	
Current smoker	9 (12.9)	11 (16.7)	4 (5.6)	
Hospitalization				
Inpatient	3 (4.3)	7 (10.6)	2 (2.8)	.1498^a^
Outpatient	67 (95.7)	59 (89.4)	70 (97.2)	
Type of influenza				
A virus	42 (60.0)	42 (63.6)	46 (63.9)	.8887^a^
B virus	28 (40.0)	24 (36.4)	26 (36.1)	
Chronic respiratory disease				
COPD	5 (7.1)	4 (6.1)	6 (8.3)	.9917^c^
Bronchial asthma	64 (91.4)	61 (92.4)	65 (90.3)	
Pulmonary fibrosis	1 (1.4)	1 (1.5)	1 (1.4)	
Body temperature				
<37°C	0 (0.0)	0 (0.0)	0 (0.0)	.1720^b^
≥37°C to < 38°C	21 (30.0)	29 (43.9)	33 (45.8)	
≥38°C to < 39°C	37 (52.9)	30 (45.5)	26 (36.1)	
≥39°C to < 40°C	10 (14.3)	7 (10.6)	11 (15.3)	
≥40°C	2 (2.9)	0 (0.0)	2 (2.8)	
Total score of 3 respiratory symptoms	5.0 ± 1.4	4.9 ± 1.4	5.0 ± 1.5	
≥5 score	45 (64.3)	43 (65.2)	44 (61.1)	.8730^a^
<5 score	25 (35.7)	23 (34.8)	28 (38.9)	
Total score of 7 influenza symptoms	12.4 ± 2.9	11.7 ± 3.1	12.4 ± 3.2	
CAT total score	18.3 ± 8.9	19.5 ± 10.0	20.0 ± 8.2	
CAT 8 items, score				
Cough	2.9 ± 1.3	3.0 ± 1.4	3.0 ± 1.1	
Phlegm	2.2 ± 1.5	2.2 ± 1.6	2.5 ± 1.5	
Chest tightness	2.3 ± 1.4	2.4 ± 1.6	2.5 ± 1.4	
Breathlessness	2.5 ± 1.6	2.6 ± 1.8	2.9 ± 1.5	
Activities	2.0 ± 1.6	2.1 ± 1.8	2.0 ± 1.7	
Confidence to get out	1.4 ± 1.6	1.9 ± 1.7	1.6 ± 1.5	
Sleep	2.0 ± 1.7	2.1 ± 1.7	2.2 ± 1.6	
Energy	3.0 ± 1.5	3.2 ± 1.5	3.2 ± 1.4	
Virus titer, logTCID_50_	5.63 ± 2.04	5.38 ± 2.21	5.44 ± 2.13	

Note

Values are n (%) or mean ± standard deviation.

Abbreviations: CAT, Chronic Obstructive Pulmonary Disease Assessment Test; COPD, chronic obstructive pulmonary disease; ITT, intent‐to‐treat; TCID_50_, 50% tissue culture infectious dose.

^a^ Fisher exact test.

^b^ Kruskal‐Wallis test.

^c^ Pearson's chi‐squared test.

### 3.2 Mean change from baseline in total and individual item CAT scores

**FIGURE 1:**
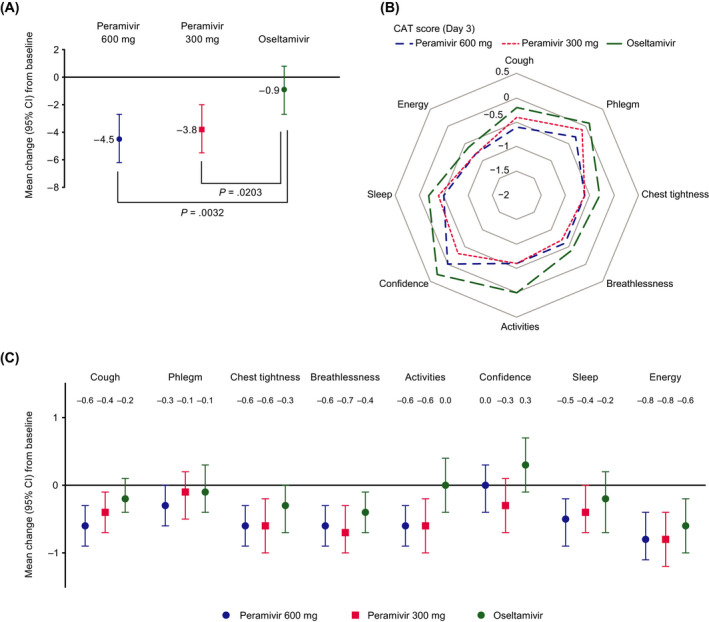
Mean change from baseline to Day 3 in (A) total score of Chronic Obstructive Pulmonary Disease Assessment Test (CAT), (B and C) score of eight CAT items. Values are mean (± 95% confidence interval [CI] in panels A and C)

Both peramivir treatment regimens reduced the total CAT score to a significantly greater extent than oseltamivir (peramivir 600 mg vs oseltamivir, *P* = .0032; peramivir 300 mg vs oseltamivir, *P* = .0203) at Day 3 (Figure ). The mean change from baseline in the total CAT score at Day 3 was −4.5, −3.8, and −0.9 in the peramivir 600 mg, peramivir 300 mg, and oseltamivir groups, respectively. The mean change from baseline was −8.8, −9.9, and −8.1 at Day 7 and −12.9, −14.5, and −12.5 at Day 14 in the peramivir 600 mg, peramivir 300 mg, and oseltamivir groups, respectively, with no statistically significant difference between groups. At Day 3, cough and phlegm were numerically more improved in the peramivir 600 mg group compared with other groups (Figure 1B,C). Breathlessness and confidence were slightly more improved in the peramivir 300 mg group compared with other groups. The scores for phlegm, activities, and confidence were not improved in the oseltamivir group. The largest difference between the peramivir and oseltamivir groups was seen in the change in activities score. At Day 14, all symptoms were improved in all three treatment groups. Cough, activities, confidence, sleep, and energy were numerically more improved in the peramivir 300 mg group than in the other groups.

### 3.3 Mean change in the total severity score of seven influenza symptoms

**FIGURE 2:**
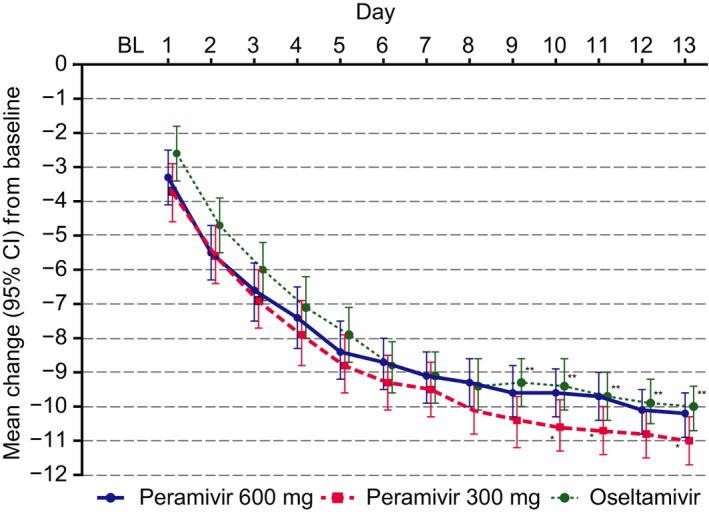
Mean change from baseline (BL) to Day 13 in total score of seven symptoms. Values are mean and 95% confidence interval (CI). *P < .05 (peramivir 600 mg vs peramivir 300 mg). **P < .05 (peramivir 300 mg vs oseltamivir)

The total severity score of seven symptoms in the three treatment groups decreased over the 14‐day period and decreased by half in about 3 days (Figure 2). The mean change from baseline in the total score of seven symptoms was greater for peramivir 300 mg compared with oseltamivir at Days 9‐13 and compared with peramivir 600 mg at Days 10, 11, and 13.

### 3.4 Time to alleviation of three respiratory symptoms

**FIGURE 3:**
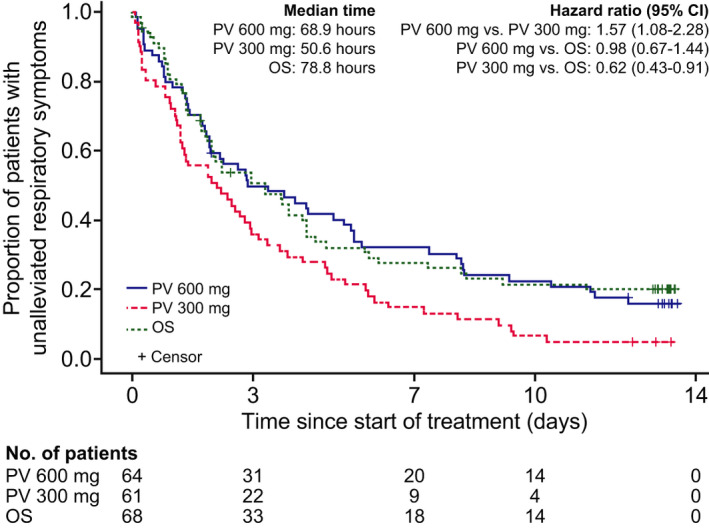
Time to alleviation of three respiratory symptoms, estimated using a Kaplan‐Meier survival curve. Medians and 95% confidence intervals (CIs) were calculated by the Greenwood method. Hazard ratios and 95% CIs were determined using a Cox proportional hazard model with prognostic factors as covariates. OS, oseltamivir; PV, peramivir

The median time to alleviation of three respiratory symptoms was 68.9, 50.6, and 78.8 hours in the peramivir 600 mg, peramivir 300 mg, and oseltamivir groups, respectively, with significant differences in the analysis of peramivir 600 mg vs peramivir 300 mg (HR 1.57; *P* = .0191) and peramivir 300 mg vs oseltamivir (HR 0.62; *P* = .0141) (Figure 3). Based on the RMST up to Day 14, the time to alleviation of three respiratory symptoms was significantly shorter with peramivir 300 mg (80.7 hours) than with peramivir 600 mg (126.5 hours; *P* = .0136) or oseltamivir (123.4 hours; *P* = .0209).

### 3.5 Time to alleviation of seven influenza symptoms

**FIGURE 4:**
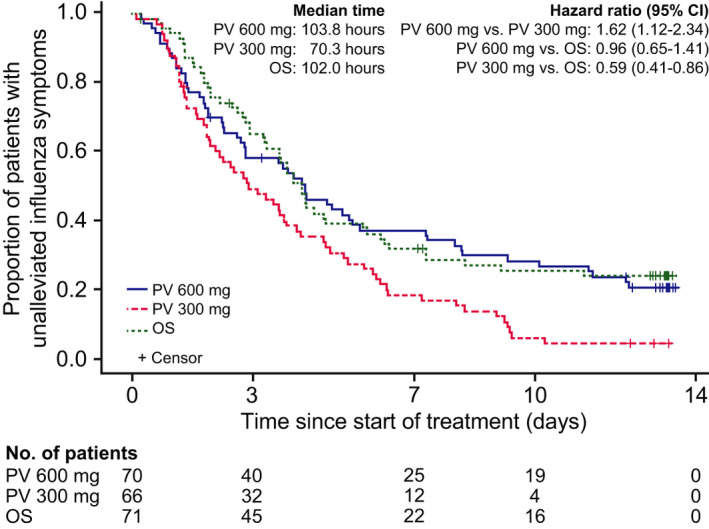
Time to alleviation of seven influenza symptoms, estimated using a Kaplan‐Meier survival curve. Medians and 95% confidence intervals (CIs) were calculated by the Greenwood method. Hazard ratios and 95% CIs were determined using a Cox proportional hazard model with prognostic factors as covariates. OS, oseltamivir; PV, peramivir

The median time to alleviation of seven influenza symptoms was 103.8, 70.3, and 102.0 hours in the peramivir 600 mg, peramivir 300 mg, and oseltamivir groups, respectively, with significant differences in the analysis of peramivir 600 mg vs peramivir 300 mg (HR 1.62; *P* = .0105) and peramivir 300 mg vs oseltamivir (HR 0.59; *P* = .0057) (Figure 4). Based on the RMST up to Day 14, the time to alleviation of seven influenza symptoms was significantly shorter with peramivir 300 mg (99.8 hours) than with peramivir 600 mg (147.1 hours; *P* = .0084) or oseltamivir (148.1 hours; *P* = .0060).

### 3.6 Time to resolution of fever

**FIGURE 5:**
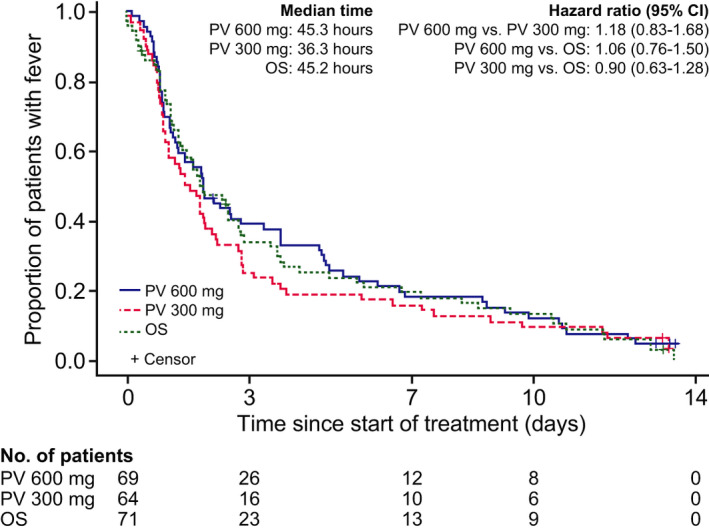
Time to resolution of fever. Hazard ratios and 95% confidence intervals (CIs) were determined using a Cox proportional hazard model with prognostic factors as covariates. OS, oseltamivir; PV, peramivir

The median time to resolution of fever was 45.3, 36.3, and 45.2 hours in the peramivir 600 mg, peramivir 300 mg, and oseltamivir groups, respectively, with no significant differences in the analysis (Figure 5). Based on the RMST up to Day 14, the time to resolution of fever was shorter with peramivir 300 mg (74.9 hours) than with peramivir 600 mg (91.9 hours; *P* = .2999) or oseltamivir (87.4 hours; *P* = .4394), with no significant difference between groups.

### 3.7 Incidence rate of exacerbation events

The incidence rate of exacerbation events within 2 weeks from first administration ranged from 15.2% to 20.8%, with no significant difference between groups (Table 2). Most exacerbation events were related to respiratory system disease (asthma, coughing, etc).

**TABLE 2:** Incidence rate of exacerbation events within 2 wks from Day 1 (ITT population)

	N	Exacerbation event	
		n	% (95% CI)^a^
Peramivir 600 mg	70	11	15.7 (8.1 to 26.4)
Peramivir 300 mg	66	10	15.2 (7.5 to 26.1)
Oseltamivir	72	15	20.8 (12.2 to 32.0)

	Difference in exacerbation rate	
	% (95% CI)	*P*‐value^b^
Peramivir 600 mg vs peramivir 300 mg	0.6 (−11.8 to 13.0)	.9282
Peramivir 600 mg vs oseltamivir	−4.6 (−17.4 to 8.2)	.4797
Peramivir 300 mg vs oseltamivir	−5.0 (−17.9 to 8.0)	.4596

Abbreviations: CI, confidence interval; ITT, intent‐to‐treat.

^a^ Clopper‐Pearson test.

^b^ Mantel‐Haenszel test with total score of three respiratory symptoms (≥5 or <5 score) at baseline and chronic respiratory disease as covariates.

### 3.8 Mean change from baseline to Day 3 in virus titer by influenza virus type and chronic respiratory disease

In patients with influenza type A, the mean change from baseline in virus titer at Day 3 was significantly greater in the peramivir 600 mg group than in the oseltamivir group (*P* = .0313). No significant difference between groups was seen in patients with influenza type B. In patients with bronchial asthma, the mean change from baseline in virus titer at Day 3 was significantly greater in the peramivir 600 mg group than in the oseltamivir group (*P* = .0466) (Table 3).

**TABLE 3:** Mean change in virus titer from baseline to Day 3 by influenza type and in patients with bronchial asthma (ITT population)

Variable	n	Mean	SD	Difference, *P*‐value^a^		
				600 mg vs 300 mg	600 mg vs OS	300 mg vs OS
Influenza type A						
Peramivir 600 mg	40	−4.29	1.84	.3052	.0313	.3434
Peramivir 300 mg	42	−3.73	2.16			
Oseltamivir	44	−3.43	1.99			
Influenza type B						
Peramivir 600 mg	27	−2.94	3.01	.9652	.6893	.5605
Peramivir 300 mg	23	−3.06	2.63			
Oseltamivir	25	−2.45	2.52			
Bronchial asthma						
Peramivir 600 mg	61	−3.80	2.47	.4078	.0466	.3220
Peramivir 300 mg	61	−3.50	2.29			
Oseltamivir	63	−3.09	2.31			

Abbreviations: ITT, intent‐to‐treat; OS, oseltamivir; SD, standard deviation.

^a^ Van Elteren test was conducted with total score of three respiratory symptoms (≥5 or <5) at baseline and chronic respiratory disease as covariates.

## 4 DISCUSSION

The work presented here is an additional analysis of our previous study^22^ and is the first study to compare the efficacy of intravenous peramivir (600 mg repeat‐dose or 300 mg single‐dose) and oral oseltamivir on CAT score, severity and time to alleviation of influenza symptoms, time to resolution of fever, incidence rate of exacerbation events, and virus titer in influenza patients with chronic respiratory disease. Compared with oseltamivir, both doses of peramivir were associated with a significantly greater improvement in total CAT score—an assessment of HRQoL—at Day 3, indicating an early response. Cough and phlegm were numerically more improved in the peramivir 600 mg group than in the other groups. Peramivir 300 mg was associated with faster time to alleviation of influenza symptoms, including respiratory symptoms, compared with oseltamivir. These results support the use of peramivir as an effective medication for influenza patients with chronic respiratory disease.

This is the first study to find a rapid improvement in the CAT, including cough, in influenza patients with chronic respiratory disease treated with peramivir. Cough was improved at Day 3 in the peramivir 600 mg group compared with the other treatment groups; the improvement may be related to the rapid reduction of virus titer seen with peramivir. Rapid improvement of cough with peramivir may lower the risk of asthma attack or exacerbation and improve patients' general activities. Notably, there was no improvement of phlegm, confidence, or activities at Day 3 with oseltamivir. Of these, the most marked difference between oseltamivir and peramivir was in the activities score. These findings suggest that alleviation of both asthma symptoms (cough and breathlessness) and influenza symptoms (quality of sleep and activities) contributed to improvement of overall HRQoL. The exacerbation of asthma (virus‐induced asthma or post‐infectious cough) can be associated with various types of respiratory virus^4^ and higher viral load. Possible mechanisms underlying cough include damage to the respiratory epithelium induced by a respiratory virus, with subsequent sensory neuron stimulation,^23^ and cytokine‐induced activation of the epithelium, with subsequent eosinophilic respiratory inflammation.^24^ The strong antiviral effect of peramivir may reduce viral load, thereby preventing damage to respiratory epithelial cells^7^ and prolonged cough symptoms.

The faster alleviation of symptoms with peramivir (600 mg or 300 mg) compared with oseltamivir was consistent with results from a previous observational study^13^ and other randomized trials in healthy adults,^25^, ^26^ high‐risk patients,^11^ and outpatients,^12^ as well as the primary results of this study.^22^ Peramivir 300 mg single‐dose was more effective at shortening the time with influenza symptoms, including respiratory symptoms, than peramivir 600 mg repeat‐dose or oseltamivir, again consistent with the primary findings.^22^ In contrast, in a previous randomized trial in a small number of high‐risk patients, most of whom had chronic respiratory disease, the duration of influenza illness was shorter with peramivir 600 mg/d than with peramivir 300 mg/day.^17^ There is no clear explanation why peramivir 300 mg single‐dose was more effective than 600 mg repeat‐dose for shortening time with symptoms in our study, although there may be bias related to patients in the 600 mg repeat‐dose group needing to return to the clinic on Day 2 for the second administration.

Among patients with influenza A or bronchial asthma, reduction of virus titer at Day 3 was significantly greater with peramivir 600 mg than with oseltamivir, generally consistent with other trials.^11^, ^17^ In contrast, for influenza B, there was no difference between treatments in virus titer reduction, possibly because of the smaller number of patients with influenza B infection. Nevertheless, the greater early antiviral effect of peramivir against influenza A compared with influenza B was roughly correlated with the differential effect on CATVS.^22^ NAIs are generally less effective against influenza B, which has a lower susceptibility to NAIs than the influenza A virus.^27^

This study was a randomized trial comparing two peramivir dosage regimens with oseltamivir that assessed a range of efficacy measures, including the CAT score as an indicator of HRQoL. The study population included only patients with chronic respiratory disease, who are particularly susceptible to respiratory exacerbations and prolonged cough, allowing us to focus on the effects of peramivir on these events. In addition, to assess the prevention of prolonged cough, we followed patients for 2 weeks, with daily diary entries, instead of approximately 1 week, which is typical of most influenza treatment studies. However, as an open‐label trial, there was potential for bias, especially for patient‐reported outcomes such as the CAT score. In addition, the exertion required to attend the second clinic visit in the peramivir 600 mg repeat‐dose group may have delayed these patients' recoveries. Most patients were outpatients, which limited our ability to assess early viral changes on Day 2. Finally, future studies should consider using the Leicester Cough Questionnaire for specific evaluation of prolonged cough in patients with chronic respiratory disease.^28^

## 5 CONCLUSION

This was the first study to assess HRQoL using the CAT score, as well as improvement of respiratory and other influenza symptoms, with administration of peramivir at two doses in influenza patients with chronic respiratory disease. The rapid improvement in CAT score (including cough) associated with peramivir treatment would be of potential benefit to patients with respiratory disease. In conclusion, we recommend that peramivir may be an appropriate first‐line prescription medication for influenza patients with chronic respiratory disease.

## CONFLICT OF INTEREST

MK is a steering committee member for AstraZeneca KK, Nippon Boehringer Ingelheim Co., Ltd., GlaxoSmithKline KK, and Shionogi & Co., Ltd., and has given lectures for AstraZeneca KK, Nippon Boehringer Ingelheim Co., Ltd., GlaxoSmithKline KK, Novartis Pharma KK, Sanofi KK, and Shionogi & Co., Ltd. YS is an employee of Shionogi & Co., Ltd. HT has received speaker honoraria from GlaxoSmithKline KK, KYORIN Pharmaceutical Co., Ltd., AstraZeneca KK, Shionogi & Co., Ltd., Meiji Seika Pharma Co., Ltd., Nippon Boehringer Ingelheim Co., Ltd., Sanofi KK, Novartis Pharma KK, Hisamitsu Pharmaceutical Co., Inc, and TEIJIN Pharma Limited. TB is an executive director of the Japan Physicians' Association and has received study funding support from Shionogi & Co., Ltd., Daiichi Sankyo Co., Ltd., and Pfizer Japan Inc.

## AUTHOR CONTRIBUTIONS

**Motokazu Kato:** Conceptualization (equal); Data curation (equal); Project administration (supporting); Resources (equal); Supervision (lead); Visualization (lead); Writing‐original draft (supporting); Writing‐review & editing (equal). **Yutaka Saisho:** Conceptualization (equal); Data curation (equal); Formal analysis (lead); Funding acquisition (lead); Project administration (lead); Visualization (equal); Writing‐original draft (supporting); Writing‐review & editing (equal). **Hiroshi Tanaka:** Investigation (equal); Resources (equal); Writing‐original draft (supporting); Writing‐review & editing (equal). **Takuma Bando:** Investigation (equal); Resources (equal); Writing‐original draft (supporting); Writing‐review & editing (equal).

## ETHICAL APPROVAL

The study was conducted in accordance with the Declaration of Helsinki, Ethical Guidelines for Medical and Health Research Involving Human Subjects, and the revised 2017 Clinical Trials Act. The protocol was approved by local ethical review boards.

## PATIENT CONSENT STATEMENT

All patients gave written informed consent.

### Peer Review

The peer review history for this article is available at https://publons.com/publon/10.1111/irv.12835.

## Data Availability

The data that support the findings of this study are available from the corresponding author upon reasonable request.
